# Temporal tracking of cysteine 34 oxidation of plasma albumin as a biomarker of muscle damage following a bout of eccentric exercise

**DOI:** 10.1007/s00421-024-05488-1

**Published:** 2024-04-16

**Authors:** Christopher James, Cory W. Dugan, Corrin Boyd, Paul A. Fournier, Peter G. Arthur

**Affiliations:** 1 School of Molecular Sciences, The University of Western Australia, Crawley, WA Australia; 2 School of Human Sciences (Exercise and Sport Science), The University of Western Australia, Crawley, WA Australia; 3 School of Veterinary Medicine, Murdoch University, Murdoch, WA Australia

**Keywords:** Oxidative stress, Eccentric exercise, Exercise-induced muscle damage, Thiol-oxidised albumin, Dried blood spot

## Abstract

**Purpose:**

Exercise-induced muscle damage (EIMD) results in the generation of reactive oxygen species (ROS), but little is known about the temporal profile of change in ROS post-EIMD and how ROS levels relate to the onset of and recovery from EIMD. Our primary aim was to examine the effect of EIMD on the pattern of change in the blood level of thiol-oxidised albumin, a marker of oxidative stress.

**Methods:**

Seven male participants were subjected on separate days to eccentric muscle contraction to cause EIMD or a no-exercise condition. After each session, the participants collected daily dried blood spots to measure thiol-oxidised albumin and returned to the laboratory every 2 days for the assessment of indirect markers of EIMD, namely maximal voluntary contraction (MVC), delayed onset muscle soreness (DOMS), creatine kinase (CK), and myoglobin.

**Results:**

Eccentric exercise resulted in a significant decrease in MVC and increase in DOMS, CK, myoglobin, and thiol-oxidised albumin with the latter reaching above baseline level within 24–48 h post-exercise. All the markers of EIMD returned to baseline level within 6 days post-exercise, but not the level of thiol-oxidised albumin which remained elevated for 10 days after exercise. There was a moderate correlation between changes in thiol-oxidised albumin and DOMS, but no significant relationship between any other markers of muscle damage.

**Conclusion:**

The levels of thiol-oxidised albumin increase in response to EIMD and remain elevated for several days post-exercise. The temporal pattern of change in the level of thiol-oxidised albumin suggests that this may be a useful biomarker of muscle repair post-EIMD.

## Introduction

Exercise-induced muscle damage (EIMD) generally occurs after a bout of unaccustomed physical activity or familiar exercise of unusual duration or intensity (Stozer et al. 2020; Tee et al. 2007). Exercise-induced muscle damage is attributed to overstretched sarcomeres and damaged extracellular matrix in skeletal muscle, and manifests with symptoms such as muscle soreness, muscle protein leakage into the circulation (e.g., creatine kinase and myoglobin), reduced muscle function (e.g. decreased maximal torque), inflammation, and increased generation of reactive oxygen species (ROS) (Hody et al. 2019; Owens et al. 2019; Stozer et al. 2020). The symptoms of EIMD can last for many days to several weeks, depending upon the extent of the damage and the individual’s susceptibility to EIMD (Harty et al. 2019; Owens et al. 2019; Paulsen et al. 2012; Peake et al. 2017; Sayers & Clarkson 2001; Stozer et al. 2020).

As a consequence of EIMD, inflammatory cells such as neutrophils and macrophages are recruited to the site of damage to remove debris and initiate a number of signalling cascades within the muscle myofiber and extracellular matrix to stimulate repair and remodelling (Bernard et al. 2022; Peake et al. 2017; Tidball 2005; Toumi et al. 2006). Inflammatory cells are also a source of ROS, with ROS being evident following EIMD and subsequent repair of skeletal muscle (Kozakowska et al. 2015; Lian et al. 2022; Powers & Schrager 2022). The increase in ROS following EIMD has been proposed to cause secondary muscle damage to uninjured muscle fibres via direct oxidative damage to biomolecules or indirect induction of inflammatory cytokines (Kawamura & Muraoka 2018; Paschalis et al. 2013; Powers et al. 2011). There is also evidence that the damage caused by ROS partly explains why decrements in muscle function and increased muscle soreness can persist for several days after a bout of eccentric exercise (Corr et al. 2021; Paschalis et al. 2007, 2013; Steinbacher and Eckl 2015).

Although EIMD increases the level of biomarkers of ROS, there are limited data on the temporal pattern of change in the level of ROS biomarkers after damaging exercise (Kawamura and Muraoka 2018; Margaritelis et al. 2014; Powers et al. 2011). In this context, there are also limited data relating changes in oxidative stress to other measures of damage, such as loss of muscle force, delayed onset muscle soreness (DOMS), and level of plasma creatine kinase (CK) and myoglobin. A study by Silva et al. (2010) found that protein carbonyls, a serum marker of ROS, were elevated on days 2, 4 and 7 days after a single bout of eccentric exercise targeting the biceps (Silva et al. 2010). This observation indicates that ROS generation may persist for at least 7 days beyond the initial damage caused by EMID. Tracking the temporal changes in blood biomarkers of ROS requires frequent collection of blood samples. However, this presents logistical challenges when blood samples are collected by venipuncture with the concomitant sample preparation and cold chain storage, thus maybe explaining why so few studies have examined the temporal changes in blood biomarkers of ROS. Recently, we described the assay of oxidised albumin cysteine 34 as a sensitive biomarker assay of ROS that can be performed on a low-volume blood sample collected from the fingertip (Lim et al. 2020), thus enabling repeated non-invasive blood sampling.

The assay developed by Lim et al (2020) measures the oxidative modification of plasma albumin (Lim et al. 2020; Peake et al. 2017; Powers et al. 2020; Tidball 2005). Reactive oxygen species in the plasma and extracellular space react with the single free cysteine residue on albumin, cysteine 34 (Cys34) (Lamprecht et al. 2008; Lim et al. 2020; Pieniazek et al. 2018). Cysteine 34 is a major antioxidant for reactive oxygen species in plasma, accounting for 80% of total plasma thiols (Bocedi et al. 2018). Cys34 can be oxidised to form a biologically reversible mixed disulfide derivative of cysteine or glutathione or an irreversible sulfinic or sulfonic derivative, causing a permanent loss of its antioxidant function (Bocedi et al. 2018; Pieniazek et al. 2018; Turell et al. 2009). Protein thiol groups are particularly sensitive to oxidation by ROS, and the oxidation of plasma albumin has been previously shown to be a more sensitive marker of oxidative stress than protein carbonyl, a widely used marker of oxidative protein damage (Lim et al. 2020). Given the high sensitivity of the assay for thiol-oxidised albumin Cys34 for detecting very small changes in oxidative stress, this marker may be highly effective at revealing small changes in oxidative stress post-EIMD, thus providing an effective tool for non-invasively following the daily temporal changes in the level of thiol-oxidised albumin after EIMD.

The primary aim of this study was to examine the extent to which the level of thiol-oxidised albumin is responsive to EIMD after eccentric exercise and how effective is this marker at tracking oxidative stress over an 11-day period post-EIMD. We hypothesised that the level of thiol-oxidised albumin would increase in the days following EIMD induced by eccentric exercise, with recovery from EIMD being accompanied by a concomitant decrease in the level of thiol-oxidised albumin. To test this hypothesis, the extent of EIMD was measured indirectly by assessing maximal voluntary contraction (MVC) on a dynamometer. The secondary aim of the study was to assess whether the temporal pattern of change in the level of thiol-oxidised albumin related to the temporal changes in other biomarkers of EIMD, including CK, myoglobin, and delayed onset muscle soreness.

## Materials and methods

### Participants

Seven healthy male participants [average age, 23.7 ± 4.1 years (range 19–31 years); height (stadiometer), 182.8 ± 7.7 cm (range 172–191 cm); weight (digital platform scale), 83.5 ± 4.8 kg (range 75.0–94.6 kg)] provided their informed written consent to participate in this study. Given our earlier findings that non-damaging exercise is associated with a large effect size in the change of thiol-oxidised albumin level (Lim et al. 2020), we have calculated, using the level of thiol-oxidised albumin as our primary outcome variable, that a sample size of seven participants provides enough statistical power (0.8) for our repeated measure study design to detect a two-sided effect size above 1.4 at a significance level of *p* < 0.05. All the participants had not engaged in any significant strength training for the past 6 months and were unaccustomed to high-intensity eccentric exercise. In addition, their daily life (e.g., line of work) did not include activities such as carrying or lifting heavy objects. The participants were screened to ensure that they had no pre-existing medical condition, no contraindication to exercise, or were not taking any medication or supplement affecting inflammation or oxidative stress. The participants were informed that they were allowed to participate in their normal daily activity but were requested to refrain from any intense physical exercise for the duration of the study. The Ethics Committee of the University of Western Australia approved this study (approval number 2021/ET000196), and all procedures conformed to the Declaration of Helsinki.

### Experimental design

Participants were randomly assigned to perform a series of eccentric contractions of the knee extensor, and a no-exercise control session on separate testing sessions. Each eccentric exercise session was preceded and followed by a MVC test, whereas for the control condition, the participants were subjected to a single MVC test each session. Both conditions were administered following a counterbalanced study design with at least 8 weeks between the two testing sessions. After each session, the participants returned to the laboratory every 2 days for the assessment of indirect markers of muscle damage, namely the levels or activities of CK, myoglobin, MVC, and DOMS. They also self-collected a dried blood spot sample every day for the measurement of the level of thiol-oxidised albumin.

### Familiarisation session

Prior to testing, all participants’ height, weight, and age were recorded. Participants were also familiarised with the Biodex dynamometer (Biodex System 4, Biodex Medical Systems, Inc., Shirley, NY) for MVC testing and the dried blood spot capillary blood sampling procedure for the assay of thiol-oxidised albumin to ensure correct use when provided with their sample collection kit.

### Exercise testing sessions

At least 7 days after the familiarisation session and following an overnight fast, participants attended the laboratory at 9 am to perform either an eccentric exercise protocol preceded and followed by an MVC test or a no-exercise control test consisting of an MVC test preceded by a fingerpick blood sample. The eccentric exercise protocol chosen for this study was identical to that used by Crameri et al (2007) who reported that their protocol produced significant muscle damage for up to 8 days after exercise (Crameri et al. 2007). The exercise protocol was as follows. A total of 210 maximal eccentric muscle contractions were performed using an isokinetic dynamometer (BioDex System 3, Biodex Medical Systems Inc., Shirley, NY, USA), with passive motion being used to warm-up. The range of motion for eccentric exercise was from 90 to 10 deg (0 deg = full extension). Stabilization straps were placed across the trunk, around the waist and around the mid-thigh of the leg to be tested. The exercise protocol consisted of two exercise phases: (i) 100 maximal eccentric contractions (10 sets of 10 repetitions) at a slow contraction speed (knee joint angular velocity 30 deg s^−1^); followed by (ii) 110 maximal eccentric contractions (11 sets of 10 repetitions) at a high contraction speed (knee joint angular velocity 180 deg s^−1^). At the completion of each contraction, the participant’s leg was immediately returned passively to the starting position (10° at an angular velocity, 60 deg s^−1^), and another eccentric contraction was initiated. A 30 s rest was adopted between each set, and a 5 min rest period was used between the two exercise phases. Maximal effort was achieved by verbally encouraging the participants to contract their legs as forcefully as possible during each set. A visual display of the dynamometer force readings was shown to the participants on a computer screen to encourage them to sustain maximal efforts.

After each exercise testing session, the participants returned to the laboratory at the same time (9 am) every 2 days for 10 days for the collection of a capillary blood sample, DOMS assessment, and an MVC test (Naz et al. 2017). A 10 day monitoring period was chosen to allow an appropriate examination of muscle recovery post-exercise. The participants were also supplied with a fitness tracker (Samsung Galaxy Fit, Samsung Australia) to record their physical activity levels to provide an objective means of ensuring that no intensive exercise had been performed during the 10 day study period. For the 48 h period prior to testing and during the 10 day period after each testing session, the participants were also asked to record their food intake to ensure that they did not consume excessive quantities of antioxidant-rich foods. In addition to food intake, physical activity level, and sleeping patterns were also recorded to help participants reproduce these conditions for all testing sessions (eccentric and no-exercise control). Following an 8 week washout period, the participants returned to complete the eccentric or no-exercise control session.

### Maximal voluntary contraction protocol

Following a passive warm-up using the same isokinetic dynamometer as that used for eccentric exercise, an MVC of the quadriceps was performed during an isometric knee extension at a knee joint angle of 70°. For each MVC test, the participants performed three static maximal voluntary knee extension efforts. Participants were verbally instructed to extend the knee as fast and forcefully as possible, and encouraged to maintain their effort for 3 s. A 30 s break was allowed between each attempt. During all trials, a visual display of the dynamometer force was provided to the participants on a computer screen. Trials with an initial countermovement (identified by a visible drop in the force signal) were disqualified, and a new trial was performed. The strain gauge signal and the lever arm position signal of the dynamometer were sampled at a 1000 Hz analogue-to-digital conversion rate using an external A/D converter (DT2801-A, Data Translation, Marlboro, MA, USA). For each attempt, torque was measured from the plateau region of the torque tracing curve and the best attempt for each participant was considered to be the participant’s MVC result.

### Capillary blood collection

For in-lab blood collections, about a 100 µL capillary blood sample was collected from the fingertip into a microfuge tube. The blood was centrifuged immediately at 2000 g for 3 min, and plasma was collected and divided into separate aliquots for analysis then frozen at − 80 °C. The collection of the blood for the assay of the level of thiol-oxidised albumin was performed using the at-home blood collection protocol described below.

For the at-home blood collection, the participants were provided with an at-home sampling kit that included ethanol swabs, lancets, and sample collection cards (PerkinElmer 226 Spot Saver RUO Card) stored in the presence of silica gel desiccant. Prior to testing, the participants were trained and familiarised with the capillary blood collection protocol. They were instructed to place two drops of blood from the finger onto the centre circle of each collection card. The cards were then stored in silica gel desiccant away from sunlight until analysis. For the duration of the study, the blood collection process was repeated each morning at least 15 min after waking and prior to any food intake.

In this study, all 154 self-collected dried blood spot samples were suitable for analysis. Additionally, none of the participants reported any adverse issues with the self-collection of dried blood spots performed at home (e.g., infection, excessive pain or needlestick injury).

### Plasma creatine kinase, myoglobin, and delayed onset muscle soreness (DOMS) measurements

Plasma CK levels were measured in duplicate using a CK-NAC kit (Randox Laboratories, Parramatta, NSW, Australia), and analysed using a BioTek Powerwave XS Spectrophotometer fitted with the KC4 (V34) program (BioTek Instruments). In brief, plasma was diluted in 0.1% (w/v) NaCl before being loaded into a 96-well plate. The enzyme reagent was then added, and absorbance (340 nm) was measured every minute at 37 °C. The resulting data were analysed for the rate of absorbance change over 30 min, using only the linear period of the reaction.

The levels of plasma myoglobin were quantified using commercial enzyme-linked immunosorbent assay (ELISA) kits (ELH-Myoglobin, RayBiotech). After determination of the optimal sample dilutions for the kit and linear range, the assay was performed according to the manufacturer’s instructions.

Prior to exercise and/or each MVC testing, participants used a visual analogue scale to record their level of muscle soreness in their knee extensor on a 100 mm horizontal line, while the centre of their quadriceps muscle was palpated. On this scale, “0” indicated that there was no pain and “100” indicated the highest level of pain.

### Thiol-oxidised albumin

The level of thiol-oxidised albumin was measured as previously described in Lim et al (2020) with the following modifications (Lim et al. 2020). Capillary blood samples were collected onto a PerkinElmer 226 Spot Saver RUO Card containing polyethylene glycol maleimide. Cards were stored with silica gel desiccant for drying prior to analysis. Albumin was extracted into 0.05% tween 20 in 20 mM phosphate with further binding to Cibacron Blue F3GA agarose. Albumin was eluted with 25 µL of 1.4 M NaCl in 20 mM phosphate buffer pH 7.4. Gel electrophoresis and imaging were performed as previously described by Lim et al. 2020. For the ratiometric calculation of the percentage of reduced albumin (RA), reversibly oxidised albumin (ROA), and irreversibly oxidised albumin (IOA), the following formula was used:

Percentage of reduced albumin (%RA)

$$\% RA = \frac{RA}{{\left( {ROA + IOA} \right) + RA}}.$$

Percentage of irreversible oxidised albumin (%IOA)

$$\% IOA = \frac{IOA}{{\left( {RA + ROA} \right) + IOA}}.$$

Percentage of reversibly oxidised albumin (%ROA)

$$\% ROA = 100\% - \left( {\% RA + \% IOA} \right).$$

### Statistical analyses

The primary outcome measure in this study was the level of thiol-oxidised albumin. The statistical analyses for the level of thiol-oxidised albumin and MVC were performed on the percent changes from pre-exercise baseline. Statistical analyses on DOMS, CK, and Myoglobin were performed on raw values. Significant differences between pre- and post-exercise time points were determined using GraphPad Prism software. Data were analysed using one-way ANOVA with repeated measures followed by Dunnett’s post hoc testing. The assumption of normality was checked prior to statistical analysis. Correlation analyses between the level of thiol-oxidised albumin and CK, myoglobin, and DOMS were performed on the percent changes from pre-exercise baseline using GraphPad Prism software. The level of change for thiol-oxidised albumin that constitutes a significance difference between serial samples was calculated by determining the Reference Change Value (RCV) using coefficients of variation for analytical and individual variability calculated using the absolute albumin thiol oxidation from the no-exercise condition. The RCV calculates a value as well as the associated probability to account for both analytical imprecision and within-subject biological variation (Fraser 2011). The RCV was calculated using Microsoft Excel software as described by Lund et al (2015). All data are presented as mean ± the 95% confidence interval (CI) of the population mean to better convey visually the level of inter-individual variability for each of the markers investigated in our study. Significance was set at p < 0.05.

## Results

**Fig. 1:**
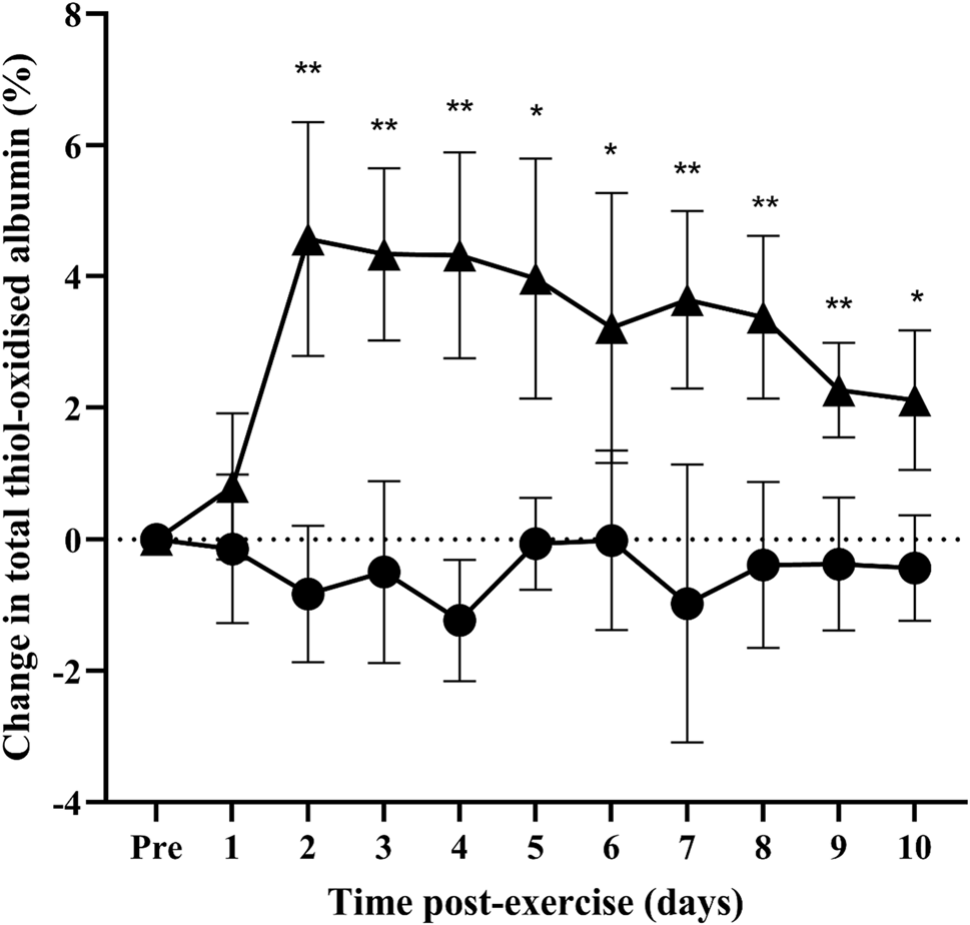
Change in the level of thiol-oxidised albumin after eccentric exercise (▲) or no exercise (●). Data are expressed relative to pre-exercise baseline values (0%) and as means ± 95% CI. ****significantly different from pre-exercise **p < 0.01, *p < 0.05. *n* = 7 participants

**Fig. 2:**
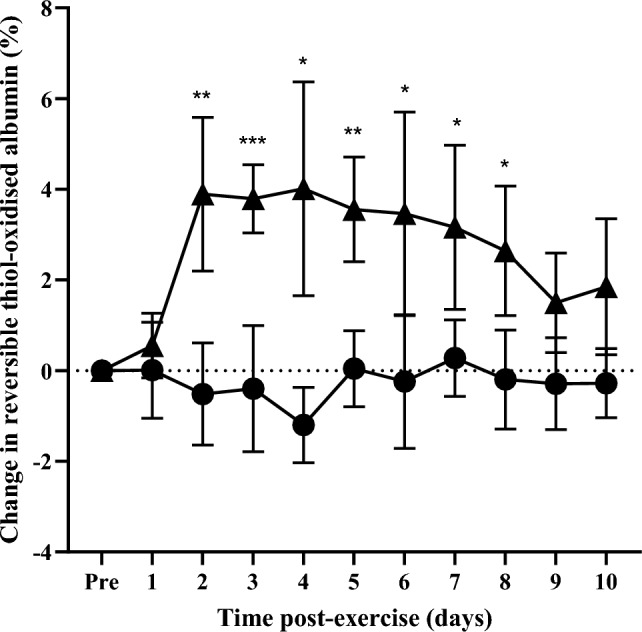
Change in reversible thiol-oxidised albumin after eccentric exercise (▲) or no exercise (●). Data are expressed relative to pre-exercise baseline values (0%) and as means ± 95% CI. n = 7 participants. ***significantly different from pre-exercise values *p* < 0.001, **p < 0.01, *p < 0.05. n = 7

**Fig. 3:**
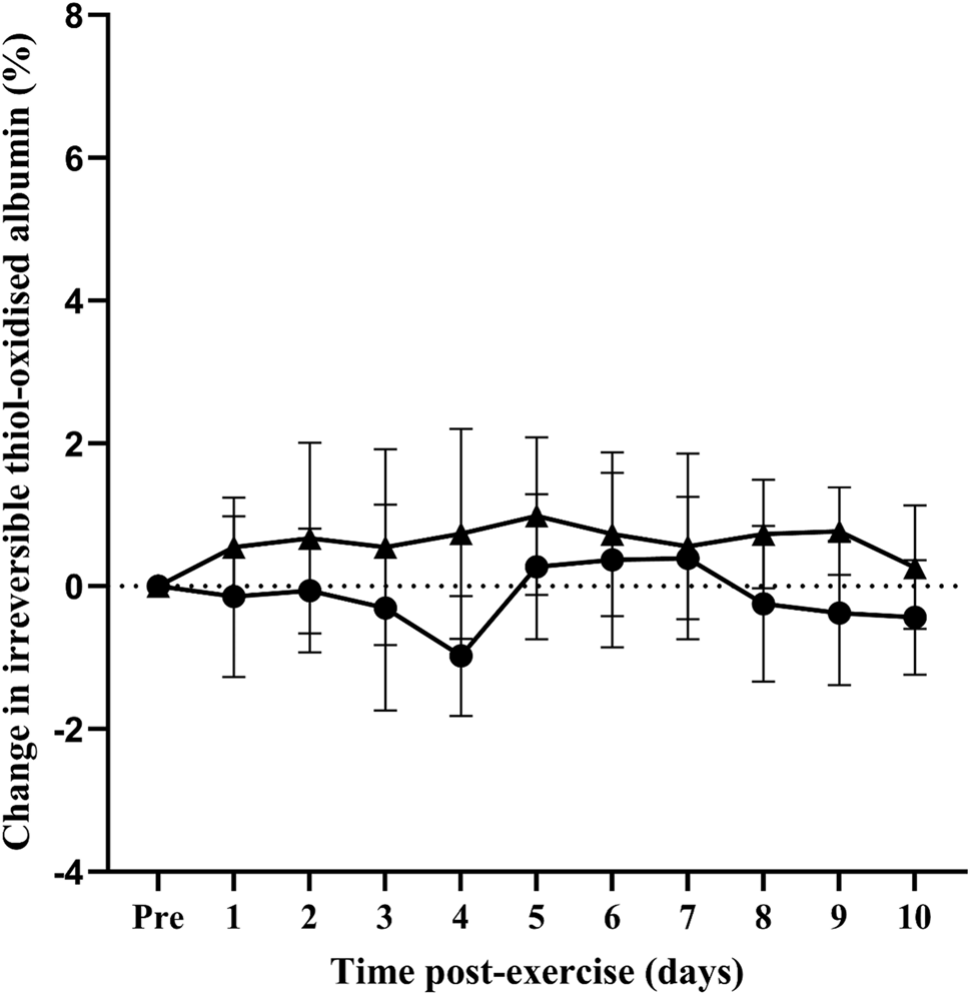
Change in irreversible thiol-oxidised albumin after eccentric exercise (▲) or no exercise (●). Data are expressed relative to pre-exercise baseline values (0%) and as means ± 95% CI. n = 7 participants***

The level of total thiol-oxidised albumin prior to eccentric exercise and no exercise was 17.4 ± 1.0% and 20.5 ± 3.8%, respectively (*n* = 7). To account for inter-individual variability, changes in the level of total, reversible, and irreversible thiol-oxidised albumin caused by eccentric exercise were expressed as change relative to pre-exercise baseline values (i.e., baseline oxidation was subtracted from all values). From pre-exercise baseline levels (i.e. 0%), the change in the level of total thiol-oxidised albumin did not differ from baseline at day 1, but was 4.5% at day 2 (Fig. 1; *p* = 0.0045) and remained elevated from day 3 until day 10 (Fig. 1; *p* = 0.015 at day 10). The change in the level of reversible thiol-oxidised albumin did not differ from baseline at day 1, but was 3.9% at day 2 (Fig. 2; *p* = 0.0078) and remained elevated from day 3 until day 8 (Fig. 2; *p* = 0.0223 at day 8). There was no significant change in the levels of irreversible thiol-oxidised albumin following eccentric exercise (Fig. 3). In the control no-exercise condition, there was no change in the level of total, reversible, or irreversible thiol-oxidised albumin for the study period (Figs. 1–3).

**Fig. 4:**
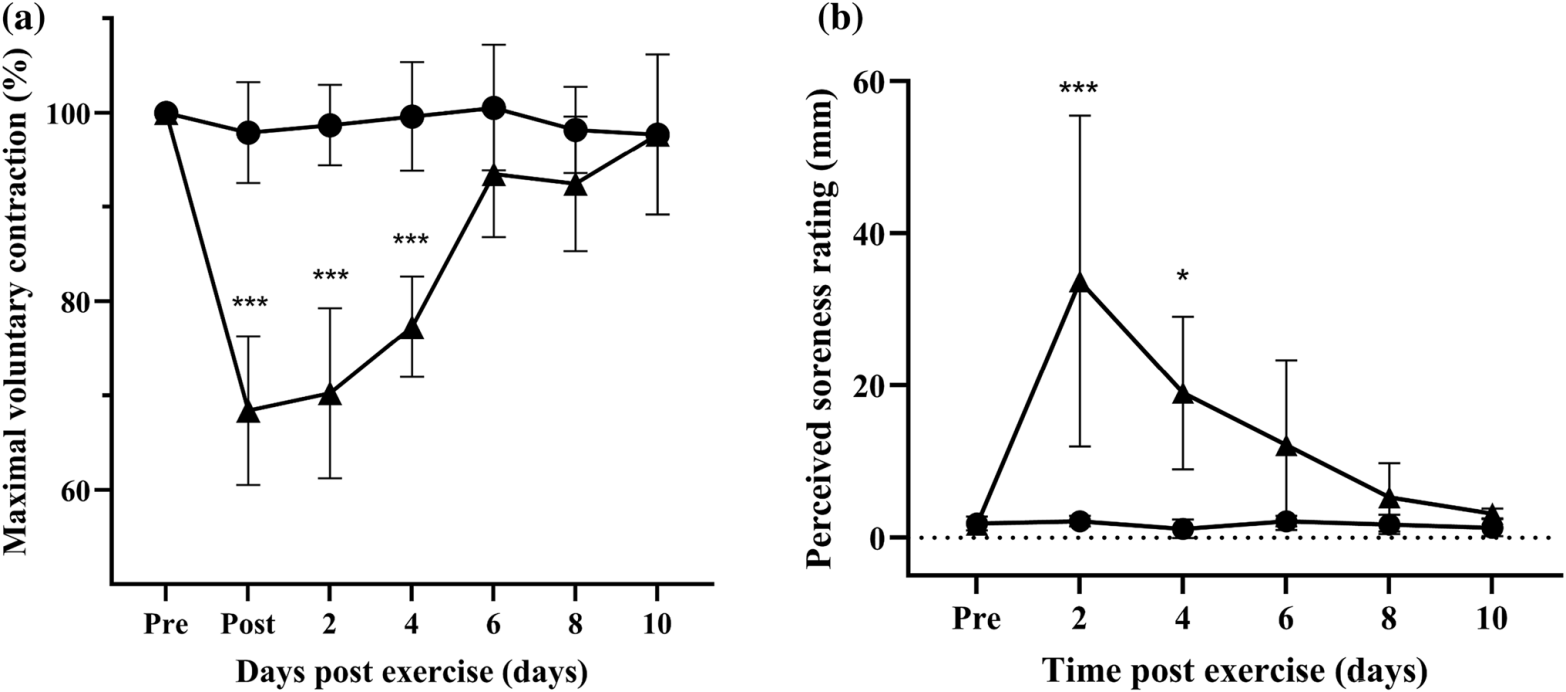
Changes in (**a**) maximal voluntary contraction (MVC) torque and (**b**) perceived delayed onset muscle soreness (DOMS) after eccentric exercise (▲) or no exercise (●). Data are expressed as means ± 95% CI and relative to pre-exercise values for MVC (100%) and in absolute terms for perceived soreness. ***significantly different from pre-exercise values *p* < 0.001, * *p* < 0.05. n = 7 participants

In response to eccentric muscle contraction, MVC decreased by 31% immediately post-exercise and remained 29% and 22% below baseline MVC on days 2 and 4, respectively (*p* < 0.0001 for each time point). Maximal voluntary contraction returned to baseline level by day 6 (Fig. 4a). For the control no-exercise condition, there was no change in MVC for the whole study period (Fig. 4a).

In response to eccentric muscle contraction, DOMS levels increased significantly on day 2 (*p* < 0.0001) and remained elevated on day 4 (*p* = 0.04), with DOMS values being no longer significantly different from pre-exercise level by day 6 (Fig. 4b). The DOMS response to eccentric exercise was variable, with the participants recording post-exercise soreness ranging from 18 up to 78 mm (100 mm = maximum). For the control no-exercise condition, there was no change in DOMS level for the whole study period (Fig. 4b).

**Fig. 5:**
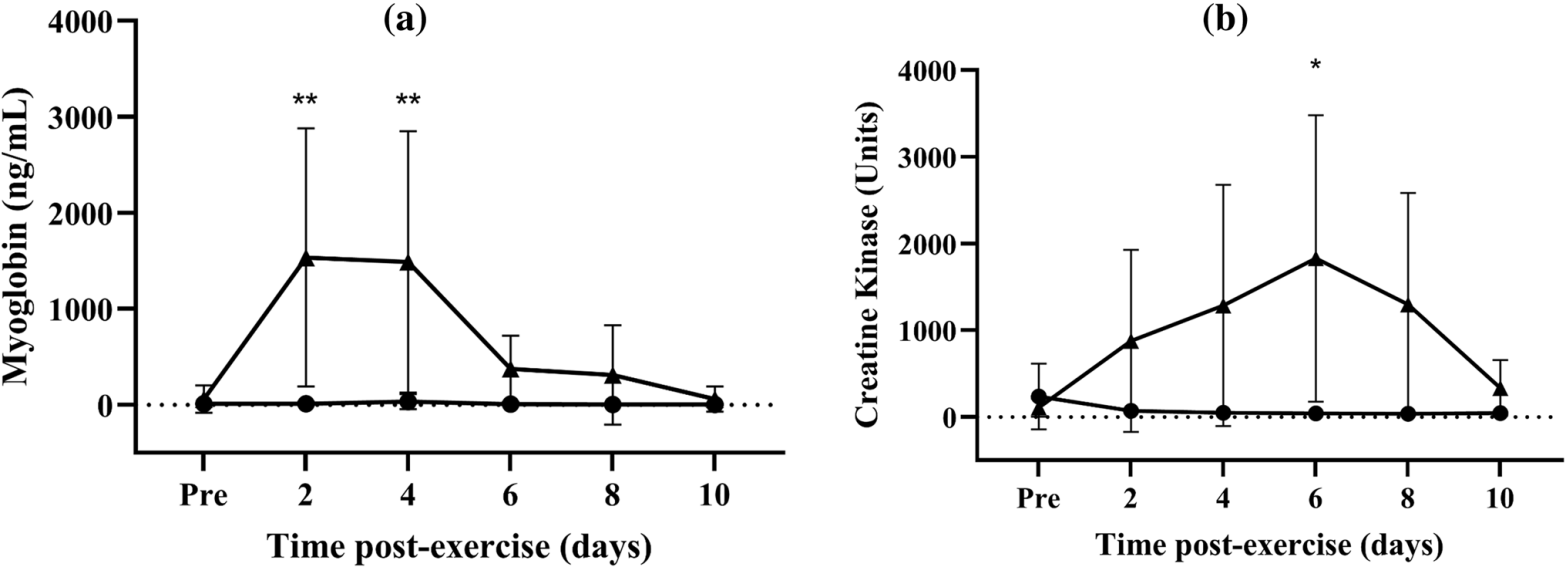
Changes in (**a**) myoglobin concentrations (ng/mL) and (**b**) creatine kinase activity (units) after eccentric exercise (▲) or no exercise (●). Data are expressed as means ± 95% CI. **significantly different from pre-exercise *p* < 0.01, *p < 0.05. *n* = 7 participants

In response to eccentric muscle contractions, the levels of plasma myoglobin increased 22-fold from baseline on days 2 (Fig. 5a; *p* = 0.03) and 4 (*p* = 0.03), returning to pre-exercise level on day 6. The levels of CK (Fig. 5b) were significantly above baseline only at 6 days after exercise, increasing 17-fold relative to baseline (*p* = 0.047). For the control no-exercise condition, there was no change in the levels of plasma myoglobin and CK for the whole study period (Fig. 5a, b).

**Fig. 6:**
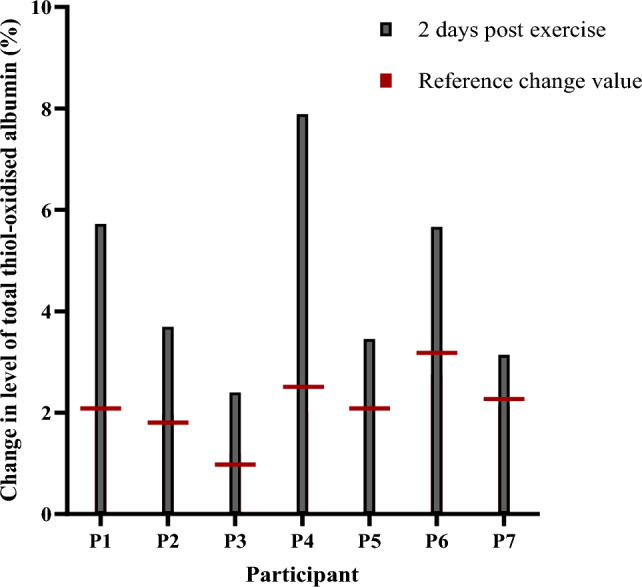
Reference change value for each participant. Change in level of albumin thiol oxidation from baseline at 2 days post-exercise for each participant (bars). Reference change value for each individual participant is shown (horizontal line)

An RCV was calculated for each participant to determine the change in the level of thiol-oxidised albumin that is accounted for by biological and analytical variability at 95% confidence after eccentric exercise (Fig. 6). A mean change from baseline of at least 2% in the level of thiol-oxidised albumin corresponded to a significant change associated with eccentric exercise. The change in the level total and reversible thiol-oxidised albumin was above the RCV cut-off for all participants at 2 days after exercise (Fig. 6).

The change in the level of total thiol-oxidised albumin from pre-exercise baseline (0%) at days 2, 4, 6, 8, and 10 post-eccentric exercise was positively correlated with DOMS (Table 1; *R*^2^ = 0.187, *p* = 0.01). Similarly, the change in the level of reversible thiol-oxidised albumin from pre-exercise baseline (0%) at days 2, 4, 6, 8, and 10 post-eccentric exercise was correlated with DOMS (Table 2; *R*^2^ = 0.19, *p* = 0.009). There was no correlation between the level of thiol-oxidised albumin and creatine kinase, myoglobin, or MVC at days 2, 4, 6, 8, and 10 post-eccentric exercise.

**Table 1:** Correlation of changes in total thiol-oxidised albumin with the muscle damage biomarkers creatine kinase (CK), myoglobin, delayed onset muscle soreness (DOMS), and maximal voluntary contraction (MVC) at 2, 4, 6, 8, and 10 days post-exercise

Damage biomarker	*P* value	*R*	*R* squared
DOMS	0.01*	0.426	0.187
CK	0.73	0.05	0.003
Myoglobin	0.20	0.21	0.048
MVC	0.23	0.27	0.061

*Correlation is significant at the 0.05 level (2-tailed)

**Table 2:** Correlation of changes in reversible thiol-oxidised albumin with the muscle damage biomarkers creatine kinase (CK), myoglobin, delayed onset muscle soreness (DOMS), and maximal voluntary contraction (MVC) at 2, 4, 6, 8, and 10 days post-exercise

Damage biomarker	*P* value	*R*	*R* squared
DOMS	0.009*	0.435	0.191
CK	0.47	0.12	0.015
Myoglobin	0.15	0.24	0.051
MVC	0.14	-0.27	0.060

*Correlation is significant at the 0.05 level (2-tailed)

## Discussion

The aim of this study was to examine if the levels of thiol-oxidised albumin are responsive to EIMD and whether post-EIMD changes in the level of thiol-oxidised albumin are closely associated with other biomarkers of EIMD. Here, we found that the level of total and reversible thiol-oxidised albumin increased within 24–48 h in response to EIMD, peaking 48 h after EMID and remaining elevated for at least 8–10 days after exercise. Thiol-oxidised albumin is thus potentially useful in detecting EMID. However, the temporal pattern of response in the change of total/reversible thiol-oxidised albumin in the days following EIMD related only weakly with DOMS and did not relate significantly with other markers of muscle damage (MVC, CK, and myoglobin). These different patterns of changes in markers of EIMD might reflect different mechanistic pathways whereby muscle damage and repair affect those markers.

It is unclear why albumin Cys34 was reversibly oxidised but not irreversibly oxidised in response to EIMD. The absence of significant changes in the level of irreversible thiol-oxidised albumin may suggest that ROS were not directly oxidising Cys34 or that the rate at which albumin was irreversibly oxidised was matched by the rate at which irreversible thiol-oxidised albumin was eliminated by the liver (Iwao et al. 2006). It should be noted that instead of direct oxidation by ROS, Cys34 can also be oxidised indirectly to the disulfide form via a thiol exchange reaction with both cystine (oxidised cysteine) and glutathione disulfide, both present in plasma (Anderson 1998; Anderson and Meister 1980; Giustarini et al. 2016; Sen and Packer 2000). In this context, albumin is the predominant source of thiol groups in plasma and could be acting as an antioxidant to regenerate cysteine and glutathione (Bocedi et al. 2018; Turell et al. 2013). Further research is required to establish whether this interpretation is valid.

The ultrastructural analysis of muscles post-EIMD has the potential to provide an effective approach to elucidate some of the molecular mechanisms leading to the oxidation of albumin Cys34. However, tracking the temporal pattern of change in muscle ultrastructure after a bout of EIMD would require multiple muscle biopsies, a highly invasive procedure. As a proxy for EIMD, MVC is regarded as a reliable technique to track muscle recovery from damaging exercise (Chalchat et al. 2022; Damas et al. 2016; Morton et al. 2005; Nosaka and Clarkson 1995; Peake et al. 2017). However, we found that the level of thiol-oxidised albumin remained elevated for longer than the duration of the MVC impairment and for this reason thiol-oxidised albumin may better track recovery from EIMD. Indeed, the eccentric exercise protocol chosen for this study was identical to that used by Crameri et al. (2007) who found that significant muscle damage and inflammation was evident for at least 8 days post-EIMD, despite the return of MVC to pre-exercise levels by day 6 (Crameri et al. 2007). Since we also found that MVC returned to basal level earlier than the level of total/reversible thiol-oxidised albumin, our findings suggest that the level of thiol-oxidised albumin Cys34 in blood may be better than MVC at tracking the ongoing muscle repair, damage, and inflammation that occurs after recovery from MVC. These findings also suggest that an elevated level of thiol-oxidised albumin is likely associated with muscle repair processes, rather than directly with processes causing loss and recovery of force generation.

The observation that it took between 24 and 48 h for the level of thiol-oxidised albumin to peak after eccentric exercise suggests that muscle damage is not an immediate source of ROS which acts to increase the thiol oxidation of albumin. These findings are consistent with those of others. Only two prior studies have investigated the response of oxidative stress markers for up to 7 days after a bout of eccentric exercise, with blood samples being collected on days 1, 2, 3, 4, and 7 post-exercise for analysis of protein carbonyl levels. Silva et al (2010) found that protein carbonyl levels were significantly elevated 4 and 7 days, but not 2 days, post-eccentric exercise of the biceps (Silva et al. 2010). Similarly, Nikolaidis et al (2007) found that the levels of protein carbonyls were elevated 2, 3, and 4 days after a bout of eccentric exercise of the knee extensors but not 1 day after exercise (Nikolaidis et al. 2007). These observations support the concept that the increase in ROS after eccentric exercise is associated with the repair phase rather than directly with muscle damage and loss of force generation.

A potential source of ROS during the repair phase is the inflammatory cells. Although neutrophils can release both hydrogen peroxide and highly reactive hypochlorous acid into the extracellular environment (Aratani 2018; Peake et al. 2005; Tidball 2005), they are unlikely to explain our findings. In the early response to EIMD, neutrophils are the most abundant immune cells that infiltrate the muscle, where they are thought to contribute further to muscle damage as well as initiate the removal of cellular debris (Peake et al. 2017; Tidball 2005; Toumi et al. 2006). Neutrophils reach their peak concentration at between 1 and 6 h after a damaging bout of exercise, and then rapidly decline to pre-exercise values by approximately 24 h post-exercise (Kanda et al. 2013; Peake et al. 2005; Toumi and Best 2003; Toumi et al. 2006). Given this timeline of events and considering that the level of thiol-oxidised albumin increased significantly within 24–48 h after EIMD, it is thus unlikely that neutrophils are responsible for the initial increase and sustained elevation in the level of thiol-oxidised albumin.

The macrophages involved in muscle regeneration and repair are another potential source of ROS (Canton et al. 2021; Etienne et al. 2020; Minari and Thomatieli-Santos 2022; Tidball 2005). In particular, the phagocytic macrophages, known as M1 macrophages, reach peak concentrations at approximately 2 days post-injury, whereas the nonphagocytic macrophages (M2 macrophages) peak at approximately 4 days post-injury and remain at a high level in the injured muscle for several days afterward (Brown et al. 2014; Martins et al. 2020; Minari and Thomatieli-Santos 2022; Oishi and Manabe 2018). The presence of macrophages from 24 h onward in the damaged muscles suggests that macrophage-derived ROS could be responsible for increasing the level of thiol-oxidised albumin in the days following EIMD. If so, then the increase in the level of thiol-oxidised albumin after EIMD would reflect the activity of macrophages and muscle repair mechanisms rather than direct muscle damage. Further research is required to determine whether this interpretation holds.

Changes in the level of thiol-oxidised albumin after eccentric exercise was significantly but weakly correlated with DOMS, and the temporal changes in the change in thiol-oxidised albumin did not reflect that of DOMS. Of note, the temporal pattern of change in DOMS found in this study reflects that reported by others in the literature, with DOMS typically peaking between 24 and 48 h after a single bout of eccentric exercise, and subsiding within 5 days (Hody et al. 2019; Mizumura and Taguchi 2016; Nosaka and Clarkson 1996; Peake et al. 2017). Also, the DOMS response to eccentric exercise was highly variable. Such variability is not surprising given the subjective nature of pain and it is well established that the level of DOMS varies considerably between individuals over time and intensity in response to the same damaging exercise stimulus (Al-Nakhli et al. 2012; Dannecker et al. 2003; Heiss et al. 2018; Hody et al. 2019).

The release of the intracellular proteins CK and myoglobin into the blood has also been used to assess muscle damage caused by EIMD. The release of both CK and myoglobin requires the permeabilisation of the plasma membrane (Sayers and Clarkson 2003; Sciorati et al. 2016). However, here, we found that their patterns of release into the circulation differed between these proteins. Creatine kinase increased above baseline level by day 6 post-exercise, a finding consistent with those of others who reported that CK activity peaked between 3 and 7 days following eccentric exercise before decreasing gradually over time (Baird et al. 2012; Hyldahl and Hubal 2014; Isaacs et al. 2019; Lee et al. 2017; Nosaka and Clarkson 1996). In contrast, myoglobin peaked at between days 2 and 4, a finding also consistent with those of others (Chalchat et al. 2022; Lavender & Nosaka 2008; Peake et al. 2017; Sayers and Clarkson 2003; Toft et al. 2002). Our finding of a marked inter-individual variability in plasma CK and myoglobin concentration response to eccentric exercise is a finding that has also been reported by others, and has been explained on the basis of inter-individual differences in the rate of release of myoglobin and CK into plasma (Hody et al. 2019; Sayers and Clarkson 2003; Weber et al. 2007). Such a marked variability may contribute to the poor relationship between temporal changes or CK or myoglobin relative to changes in thiol-oxidised albumin.

The marked inter-individual variability also extends to the baseline levels of thiol-oxidised albumin in our participants. We found that the baseline levels of total thiol-oxidised albumin ranged from 15 to 25%. The level of thiol-oxidised albumin in an individual likely reflects a balance between generation of ROS by a variety of metabolic pathways and the actions of antioxidants. Factors that have been shown to influence the levels of thiol-oxidised albumin include diet, disease, physical activity, and aging (Gryzunov et al. 2003; Lim et al. 2020; Oettl and Marsche 2010; Turell et al. 2009). This substantial between-subject variability and the lack of specificity toward EIMD implies that comparing the absolute levels of thiol-oxidised albumin of an individual to a population normal range, as is common practice with other blood analytes, is unlikely to be informative as a mean to detect EIMD.

An alternative approach is to use reference change value to assess objectively significant difference in serial results obtained for an individual (Fraser 2011). This approach is used when within-subject biological variation for a given marker is much smaller than between-subject variation (Fraser 2011). In our study where participant diet and activity levels were controlled, baseline levels of thiol-oxidised albumin were reproducible for each non-exercised individual over the study period. For this reason, changes in the level of individual thiol-oxidised albumin expressed relative to a pre-exercise baseline for the same individual could be adopted here to track EIMD. Using this approach, we found that the change in thiol-oxidised albumin levels was greater than the reference change value for all individuals on day 2 after exercise. Therefore, changes in the level of thiol-oxidised albumin may be a useful biomarker of EIMD. Given the limited number of participants in this study, and their lack of resistance exercise training, further work with a larger cohort of trained athletes is needed to test the validity of this concept.

## Conclusion

In summary, this study shows that the level of total/reversible thiol-oxidised albumin increases in response to EIMD, peaking 48 h after EMID and remaining elevated for at least 8–10 days after exercise. Our results together with those of others who have used the same exercise protocol also suggest that the elevated level of thiol-oxidised albumin in response to EIMD is likely associated with muscle repair processes rather than directly with processes causing loss and recovery of force generation, because the latter has been reported to return sooner to baseline than both muscle repair and thiol-oxidised albumin level (Crameri et al. 2007). Our data thus provide evidence that the level of thiol-oxidised albumin may provide a useful blood biomarker of repair following EIMD. Of note, the validity of this interpretation awaits the use of muscle biopsies in future studies to test if the changes in the level of total/reversible thiol-oxidised albumin track more closely muscle repair than recovery of muscle force. If our interpretation were to be corroborated, the use of total/reversible thiol-oxidised albumin as an indirect blood marker of muscle repair could be helpful in managing recovery and return to training or competition in athletes following a bout of damaging exercise or a muscle injury, particularly considering the ease to collect data non-invasively using serial small blood samples from the fingertips. Further work with a larger cohort of trained athletes is required to test the validity of this concept. The ability to track temporal changes in thiol-oxidised albumin could also be useful in improving our understanding of the role of ROS in other aspects of exercise, training, and competition where oxidative stress is evident.

## Data Availability

The data supporting the conclusions of this article can be made available by the authors, upon request.

## Abbreviations

EIMD: Exercise-induced muscle damage
ROS: Reactive oxygen species
DOMS: Delayed onset muscle soreness
CK: Creatine kinase
Cys34: Cysteine 34
MVC: Maximal voluntary contraction
ELISA: Enzyme-linked immunosorbent assay
RA: Reduced albumin
ROA: Reversibly oxidised albumin
IOA: Irreversibly oxidised albumin
ANOVA: Analysis of variance
RCV: Reference change value
